# The Effect of Innovation Capabilities of Health Care Organizations on the Quality of Health Information Technology: Model Development With Cross-sectional Data

**DOI:** 10.2196/23306

**Published:** 2021-03-15

**Authors:** Moritz Esdar, Ursula Hübner, Johannes Thye, Birgit Babitsch, Jan-David Liebe

**Affiliations:** 1 Health Informatics Research Group Faculty of Business Management and Social Sciences University of Applied Sciences Osnabrueck Osnabrueck Germany; 2 Institute of Health and Education New Public Health Osnabrück University Osnabrueck Germany; 3 Institute of Medical Informatics UMIT - Private University for Health Sciences, Medical Informatics and Technology Hall in Tyrol Austria

**Keywords:** organizational innovation, health information management, organizational culture, diffusion of innovation, hospital information systems, organizational change management

## Abstract

**Background:**

Large health organizations often struggle to build complex health information technology (HIT) solutions and are faced with ever-growing pressure to continuously innovate their information systems. Limited research has been conducted that explores the relationship between organizations’ innovative capabilities and HIT quality in the sense of achieving high-quality support for patient care processes.

**Objective:**

The aim of this study is to explain how core constructs of organizational innovation capabilities are linked to HIT quality based on a conceptual sociotechnical model on innovation and quality of HIT, called the IQ_HIT_ model, to help determine how better information provision in health organizations can be achieved.

**Methods:**

We designed a survey to assess various domains of HIT quality, innovation capabilities of health organizations, and context variables and administered it to hospital chief information officers across Austria, Germany, and Switzerland. Data from 232 hospitals were used to empirically fit the model using partial least squares structural equation modeling to reveal associations and mediating and moderating effects.

**Results:**

The resulting empirical IQ_HIT_ model reveals several associations between the analyzed constructs, which can be summarized in 2 main insights. First, it illustrates the linkage between the constructs measuring HIT quality by showing that the *professionalism of information management* explains the degree of *HIT workflow support* (*R²*=0.56), which in turn explains the *perceived HIT quality* (*R²*=0.53). Second, the model shows that HIT quality was positively influenced by innovation capabilities related to the top management team, the information technology department, and the organization at large. The assessment of the model’s statistical quality criteria indicated valid model specifications, including sufficient convergent and discriminant validity for measuring the latent constructs that underlie the measures of HIT quality and innovation capabilities.

**Conclusions:**

The proposed sociotechnical IQ_HIT_ model points to the key role of professional information management for HIT workflow support in patient care and perceived HIT quality from the viewpoint of hospital chief information officers. Furthermore, it highlights that organizational innovation capabilities, particularly with respect to the top management team, facilitate HIT quality and suggests that health organizations establish this link by applying professional information management practices. The model may serve to stimulate further scientific work in the field of HIT adoption and diffusion and to provide practical guidance to managers, policy makers, and educators on how to achieve better patient care using HIT.

## Introduction

### Background

Discussions on health information technologies (HITs) in research and practice have increasingly shifted from dealing with the question of *if* digital solutions are worth investing in [1,2] to questions on *how* higher degrees of successful digitalization can be achieved [3-6] and how HIT improves processes and outcomes [7-9]. Although the term HIT has been used and defined in various ways, we understand it to encompass the organization’s electronic information technologies that health care professionals use to support the care process [7]. These include, but are not limited to, electronic medical records, health information exchange systems, computerized provider order entry, clinical decision support systems, and the related hardware (excluding medical devices) and their integration with each other.

It has been repeatedly demonstrated that large health organizations often struggle to adopt high-quality and modern HIT solutions and are challenged with increasingly shorter innovation cycles of these technologies [10-15]. The fact that there is considerable variation in the adoption and quality of HIT between organizations within and across countries points to the importance of focusing on the organizations themselves in terms of their *inner* capabilities with regard to managerial skills, the promotion of HIT use, project execution, and innovation promotion [16-18]. Although a wide range of general facilitating factors of successful HIT adoption have been acknowledged in several theoretical frameworks [19-24] and various systematic literature reviews [3,12,25-28], little is known about the exact constituents of capabilities of health care organizations to innovate in particular and how they affect not only the adoption of HIT but also their quality. Insights about this relationship could prove valuable for guiding managers, policy makers, and educators toward promoting and developing organizational behavior that facilitates better HIT use, which in turn might lead to improved clinical outcomes [29].

### HIT Quality and Innovation Capabilities

HIT adoption is most often understood as the implementation, that is, the introduction of an application, and its acceptance and use in an organization and many adoption studies focus on specific functionalities or applications [12,21,27]. However, the complexity of organization-wide HIT solutions is usually far greater and requires the incorporation of many different facets of the organization’s information system [30-33]. In addition, when extending the scope from adoption to the quality of HIT, even more aspects need to be incorporated as quality requirements are typically considered to incorporate not only various technical layers (eg, data and information, functions, hardware, interoperability) to support clinical care processes but also features of information management and the perceived quality of the IT systems [17,23,34,35]. Thus, in our study, we focus on HIT quality rather than mere adoption and consider it to be composed of the following 3 principal domains: HIT information management, HIT workflow support, and perceived HIT quality:

- HIT information management encompasses the full spectrum of strategic, tactical, and operational management tasks to build and operate an organization’s information system [34,36]. Management practices are deemed to be essential preconditions for realizing the potential of HIT [37], especially those executed by the information technology (IT) department [38,39] and those that involve systematic clinical user participation [40,41].
- Finally, HIT quality manifests itself not only in the technical quality of HIT but also in the subjective assessment of the implemented IT solutions that is hereinafter referred to as the perceived HIT quality.

In addition to HIT quality, there is also little understanding about the identification and effect of the organization’s capabilities to innovate; however, as van Gemert-Pijnen et al [44] emphasize, many HIT innovations might fail as a result of disregarding the interdependencies between technology and its organizational and cultural environment. In our understanding, innovation capabilities (ICs) refer to the culture regarding HIT at various organizational levels that reflect its ability to innovate, that is, the ability to adopt new HIT solutions (or to renew the existing ones) that enhance the quality of information provision in clinical care processes. These capabilities refer to latent phenomena, that is, they are inherently difficult to capture, as they are expressions of a commonly shared attitude in social networks that leads to certain sets of corresponding behaviors [45,46]. In light of the semantic variations and inconsistent definitions of related phenomena, scholars have pointed to the need for further work to examine this construct and its measurement [47-49]. The lack of measurements also implies that there are few studies that provide empirically tested claims regarding the effect of an organization’s ICs on HIT adoption or quality [49,50].

### Conceptual Model and Study Objectives

Only a few theoretical frameworks incorporate the peculiarities and complexity of organization-wide HIT solutions in a way that allows for an assessment of its quality and success [23,24,34]. Others acknowledge the facilitating role of domains comparable with ICs [19,20]; however, there is no framework that puts the spotlight on the interrelationship between these 2 constructs and how they might enable better information provision in the care processes. Correspondingly, there is a need for validated measurement scales within such a framework to put its implicit hypotheses into the empirical test. Although some studies have begun to derive related scale sets [51-53], they are not yet ready to measure the full picture of the relationship between the 2 domains. In addition, the few that attempted to test more complex relationships between related constructs have limitations, particularly regarding small sample sizes and rather narrow outcome measures of HIT quality [54-56].

**Figure 1:**
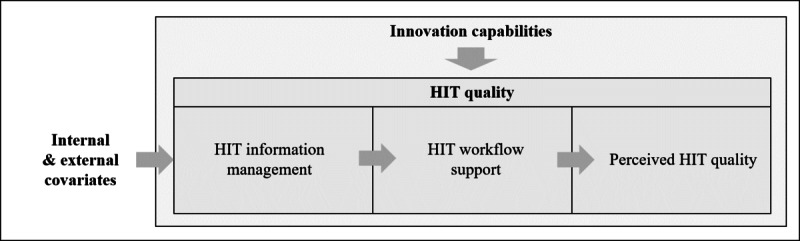
Initial conceptual innovation and quality of health information technology model of the layered relationship between innovation capabilities, health information technology quality, and covariates. HIT: health information technology.

To investigate the sociotechnical interrelationships between ICs and HIT, we propose an initial conceptual model, that is, the IQ_HIT_ (innovation and quality of HIT) model (Figure 1). It rests on the underlying assumption of a directional process of antecedents and consequences of HIT as was similarly conceptualized in studies by Leidner et al [54] and Greenhalgh et al [57]. This is reflected in the assumption that HIT information management affects the degree of HIT workflow support that then determines the perceived HIT quality. Furthermore, these domains can be assumed to be influenced by an organization’s ICs. In addition, internal structural characteristics such as the organization’s size, teaching status, and ownership as well as external influences in terms of national health policies and legal regulations need to be accounted for as possible covariates in the model, as both have been shown to be significantly associated with HIT use [58-61].

On the basis of this model, the research goal was to empirically test and explain how health organizations’ ICs are linked to HIT quality.

## Methods

### Data Collection

Serving as empirical input for the model, data from chief information officers (CIOs) as hospital representatives were obtained. Hospitals are particularly interesting because of both the complexity of their IT and their organizational environment. We chose CIOs as our target group because they have the best oversight of the entirety of the IT landscape and top management issues [62,63]. We included Austrian, German, and Swiss hospitals in our target population to control for external influences in terms of different national health policies. The questionnaire and its constructs were based on the redevelopment and refinement of previous surveys and included a total of 188 question items (Multimedia Appendices 1 and 2) [64]. The final questionnaire was pretested by 5 hospital CIOs, 10 researchers (comprising health IT experts, statisticians, and 1 psychologist), and 1 clinician to evaluate whether the question items were understandable and answerable and whether they were sufficiently precise to measure the organization’s information system. This led to some minor adjustments of item scales (response options), changes in the wording of items, and a few supplementary definitions.

Email addresses of 1669 CIOs were compiled through internet and telephone searches. The CIOs were responsible for 2324 hospitals (92% of all 2542 hospitals across Austria, Germany, and Switzerland). Data collection took place during the first half of 2017 via a web-based survey. Of the 1669 emails sent, 1499 had come through and 251 CIOs participated (17% response rate)—19 answers were discarded because of incompleteness (ie, the respondent did not finish the survey or sections were left out). The descriptive results were made available in 2018 [65], and as an incentive for participation, CIOs were offered access to a web-based benchmarking dashboard that allowed them to compare their hospital with peer groups [66].

### Modeling and Data Analysis

We applied structural equation modeling (SEM) to test various interrelationships between constructs. Specifically, we chose partial least squares structural equation modeling as it is tolerant of the use of categorical data and allows for including reflective measurement models (ie, manifest indicators *reflect* the latent construct), formative measurement models (ie, manifest indicators *form* the latent construct), and single-item scales without identification problems [67].

#### Specification of the Measurement Models

We operationalized each of the 5 domains in the conceptual model (Figure 1), with a total of 10 constructs (Table 1). All items and scales associated with these constructs are detailed in Multimedia Appendices 1 and 2.

**Table 1:** Overview of the constructs used to operationalize the domains of the conceptual innovation and quality of health information technology model.

Domains		Constructs		
**HIT^a^ quality**				
	HIT information management	Professionalism of information management	Clinical IT^b^ agents	N/A^c^
	HIT workflow support	Workflow composite score including technical descriptors and care processes	N/A	N/A
	Perceived HIT quality	Perceived HIT workflow support	Overall goodness of information provision	N/A
Innovation capabilities		Innovation capability: top management team support	Innovation capability of the information technology department	Organization-wide innovation capability
Covariates		Structural characteristics	Country	N/A

^a^HIT: health information technology.

^b^IT: information technology.

^c^N/A: not applicable.

##### HIT Quality

HIT information management was operationalized using 2 constructs. First, we applied a construct that captures the degree of professionalism of information management (PIM) in health care in terms of the regularity of 15 management key tasks and practices, as proposed by Thye et al [36]. As PIM consists of 3 latent and correlated subcomponents (strategic, tactical, and operational information management), we incorporated it as a reflective higher order model with PIM as the higher order construct and the 3 subcomponents as the lower order constructs using the repeated indicator approach [68]. Second, to reflect institutionalized user participation, we included the formal appointment of clinical IT agents as a reflective measurement model with 2 underlying items (one referring to physicians and the other one to nurses).

HIT workflow support can be theorized as being constituted by the descriptors data and information, IT functions, integration, and distribution of data and IT functions [17]. These 4 descriptors are the central building blocks of the Workflow Composite Score (WCS), an aggregated score that proved to be reliable and valid in measuring the degree of HIT supported patient care in core clinical processes [17,43,65]: ward rounds to reflect diagnostic and therapeutic decision making at the bedside, presurgery and postsurgery processes that reflect the information flow between departments, and admission and discharge as core interface processes between outpatient and inpatient care. The WCS comprises 146 items grouped along these 5 clinical processes and the 4 descriptors (Multimedia Appendix 2). We included it in the SEM analysis as a single-item scale, as its composite structure was largely predefined in previous studies [17,65].

Perceived HIT quality was measured using the 2 constructs. First, we asked the CIOs to grade the HIT workflow support (perceived HIT workflow support) in all 5 aforementioned clinical care processes separately and included the resulting indicators in a reflective measurement model. Second, we asked for a concluding assessment (single-item scale) of the overall goodness of information provision, that is, the organization’s general ability to provide the right information, at the right time, at the right place, for the right persons, and in the right quality to support patient care processes. This indicator was applied in a previous study [38].

##### Innovation Capabilities

We investigated this domain and the underlying constructs across the 2 preceding surveys [38,52]. The initial exploratory study on this topic pointed to a latent construct, represented by 5 items that describe the top management team (TMT) support and the organization-wide innovation culture with regard to HIT [52]. A second study signified that the ICs relating to the IT department could be considered as another separate component [38]. To explore the emerging constructs in greater depth, we added 9 items to capture additional details on the TMT support and the organization-wide innovation culture and 6 additional items that refer to the IT department. An exploratory factor analysis using the unweighted least squares estimation and oblique factor rotation was computed, which resulted in a 3-factor structure that reflected ICs at the TMT level (IC TMT), ICs at the IT department level (IC ITD), and ICs at the organization-wide level (IC OW). For SEM, the underlying items were then included in 3 reflective measurement models. A total of 4 items with low outer loadings (<0.50) were removed to establish sufficient convergent and discriminant validity.

##### Covariates

A total of 2 covariates were included in the model. First, to control for well-known structural characteristics, we included a formative measurement model that was composed of the hospital size (bed count) and its teaching status. Second, the country was included as a single-item scale to account for external conditions. Austrian and Swiss hospitals were pooled to obtain more balanced group sizes.

#### Specification of the Structural Model

The specifications of the structural model resulted from a step-wise build-up of testing the direct and mediated effects along the components of the conceptual model. Each step was thereby rooted in findings from studies that suggest individual linkages between the constructs, which we summarized into a set of 12 theoretical assumptions (Table 2). On the basis of these assumptions, we deduced one or more hypotheses for specifying the structural equation model paths.

**Table 2:** Theoretical assumptions and corresponding hypotheses guiding the structural model specification.

Assumption	Exemplary study
The PIM^a^ might be linked to HIT^b^ workflow support H1: PIM has a positive effect on the WCS^c^	Ammenwerth et al (2006) [69], Avgar et al (2012) [70], Bradley et al (2012) [71], Winter et al (2011) [72]
Formal participation in terms of the appointment of clinical IT^d^ agents might results from PIM practices and might lead to better HIT workflow support H2: The effect of PIM on the WCS is partly mediated by clinical IT agents	Cresswell and Sheikh (2013) [12], Liebe et al (2018) [42], Potts et al (2011) [73], Sligo et al (2017) [26]
There likely is a direct link between the technical and the perceived quality of HIT workflow support H3: The WCS has a positive effect on the perceived HIT workflow support H4: The WCS has a positive effect on the overall goodness of information provision	Hadji and Degoulet (2016) [74], Hübner (2015) [75], Yusof et al (2008) [23]
The perceived quality of HIT is likely linked to the perceived goodness of information provision H5: Perceived HIT workflow support has a positive effect on the overall goodness of information provision	Gorla et al (2010) [76], Suki (2012) [77]
A top management team that is capable and willing to innovate might facilitate an innovation-friendly culture throughout the organization, including the IT department H6: Innovation capability: top management team support has a positive effect on organization-wide innovation capability H7: Innovation capability: top management team support has a positive effect on the innovation capability of the IT department	Abdekhoda et al (2015) [78], Carpenter et al (2004) [79], Laukka et al [80]
The tasks and procedures that manifest PIM might also be facilitated by an innovation-friendly top management team H8: Innovation capability: top management team support has a positive effect on PIM	Bradley et al (2012) [71], Liebe et al (2018) [81], Weintraub and McKee (2019) [82]
Innovation capabilities of the top management team and the IT department might determine the degree of HIT workflow support H9: Innovation capability: top management team support has a positive effect on the WCS H10: Innovation capability of the IT department has a positive effect on the WCS	Esdar et al (2018) [38], Paré et al (2020) [56], Leidner et al (2010) [54]
The ability of the IT department to innovate might be linked to information management practices H11: Innovation capability of the IT department has a positive effect on PIM	Liebe et al (2017) [83], Watts and Henderson [84]
HIT quality might be a function of the organization-wide climate toward IT. Such climate might also facilitate a stronger effect of the technical HIT quality (ie, the WCS) on the perceived quality of information provision H12: Organization-wide innovation capability has a positive effect on the WCS H13: Organization-wide innovation capability has a positive effect on the perceived HIT workflow support H14: Organization-wide innovation capability has a positive effect on the overall goodness of information provision H15: Organization-wide innovation capability positively moderates the relationship between the WCS and the overall goodness of information provision	Caccia-Bava et al (2006) [45], Gagnon et al (2012) [85], Taylor et al (2015) [86], Vest et al (2019) [50]
Structural characteristics might be linked to HIT quality, possibly also to the TMT’s capabilities to innovate H16: Structural characteristics have a positive effect on the WCS H17: Structural characteristics have a positive effect on PIM H18: Structural characteristics have a positive effect on the innovation capability: top management team support	DesRoches et al (2012) [58], Fadol et al (2015) [87], Kruse et al (2014) [88], Troilo et al (2014) [89]
Compared with Germany, hospitals from Austria and Switzerland exhibit higher degrees of HIT workflow support and a more pronounced culture toward innovation H19: Country has a positive effect on the WCS H20: Country has a positive effect on organization-wide innovation capability H21: Country has a positive effect on the innovation capability of the IT department	Esdar et al (2018) [38], Haux et al (2018) [90], Hübner et al (2010) [91], Hüsers et al (2017) [49], Naumann et al (2019) [11]

^a^PIM: professionalism of information management.

^b^HIT: health information technology.

^c^WCS: Workflow Composite Score.

^d^IT: information technology.

#### Parameter Estimations and Model Assessment

We applied partial least squares structural equation modeling using SmartPLS version 3 [92]. The measurement models were assessed for internal consistency using Cronbach α and composite reliability. Convergent and discriminant validity was evaluated according to the height of the outer loadings, the average variance extracted, the Fornell-Larcker criterion, and the Heterotrait-Monotrait ratio.

Inner variance inflation factor values were used to test for collinearity within the structural model. Path coefficients and mediation effects were evaluated based on the direct, total, and indirect effects as well as on f² effect sizes with *P* values and 95% CIs obtained from 10,000 bootstrap replications. Besides the *R²* values for the endogenous latent variables, we used blindfolding to obtain Stone-Geisser Q² values to determine the cross-validated predictive relevance of the exogenous constructs.

## Results

### Descriptive Statistics

The sample consisted of data from 232 hospitals, most of which were from Germany (Table 3), which corresponds to the higher baseline number of German hospitals. The participating hospitals were rather large, with an average size of 492 (SD 239) beds, and many (112/232, 48.3%) were in public ownership. Nevertheless, hospitals from all relevant demographic categories were included in the sample. The WCS, as the central measure of HIT workflow support in our model, showed an overall mean value of 56 (SD 14) points (ranging between 0 and 100 points; Multimedia Appendix 3). The mean values and SD of the remaining constructs are shown in Multimedia Appendix 1.

**Table 3:** Demographic characteristics of participating hospitals (N=232).

Characteristics			Value
**Country, n (response rate in %)**			
	Austria	14 (8.8)	
	Germany	205 (18.3)	
	Switzerland	13 (11.3)	
**Ownership, n (% in sample)**			
	For-profit	42 (18.1)	
	Nonprofit	78 (33.6)	
	Public	112 (48.3)	
**Teaching status, n (% in sample)**			
	Major teaching hospital	22 (9.5)	
	Minor teaching hospital	101 (43.5)	
	Nonteaching hospital	109 (47.0)	
**Member of a hospital group, n (% in sample)**			
	Yes	140 (60.3)	
	No	92 (39.7)	
Number of beds, mean (SD)			491.9 (238.5)

### Structural Equation Model

The parameters assessing the measurement models pointed to valid specifications of the reflective models as well as the formative model in terms of convergent validity and internal consistency (Multimedia Appendices 4 and 5). In addition, sufficient discriminant validity was established according to the Fornell-Larcker criterion assessment, as indicated by the Heterotrait-Monotrait ratios of the correlations that were all below the recommended threshold value of 0.85 [93] (Multimedia Appendix 6). No collinearity was found in the structural model, as all the inner variance inflation factor values ranged within the limits of 0.20 and 4. Moreover, the Stone-Geisser Q² values of the endogenous variables indicate a good out-of-sample predictive power of the path model, especially with regard to the WCS (Q²=0.38) and the overall goodness of information provision (Q²=0.40).

**Figure 2:**
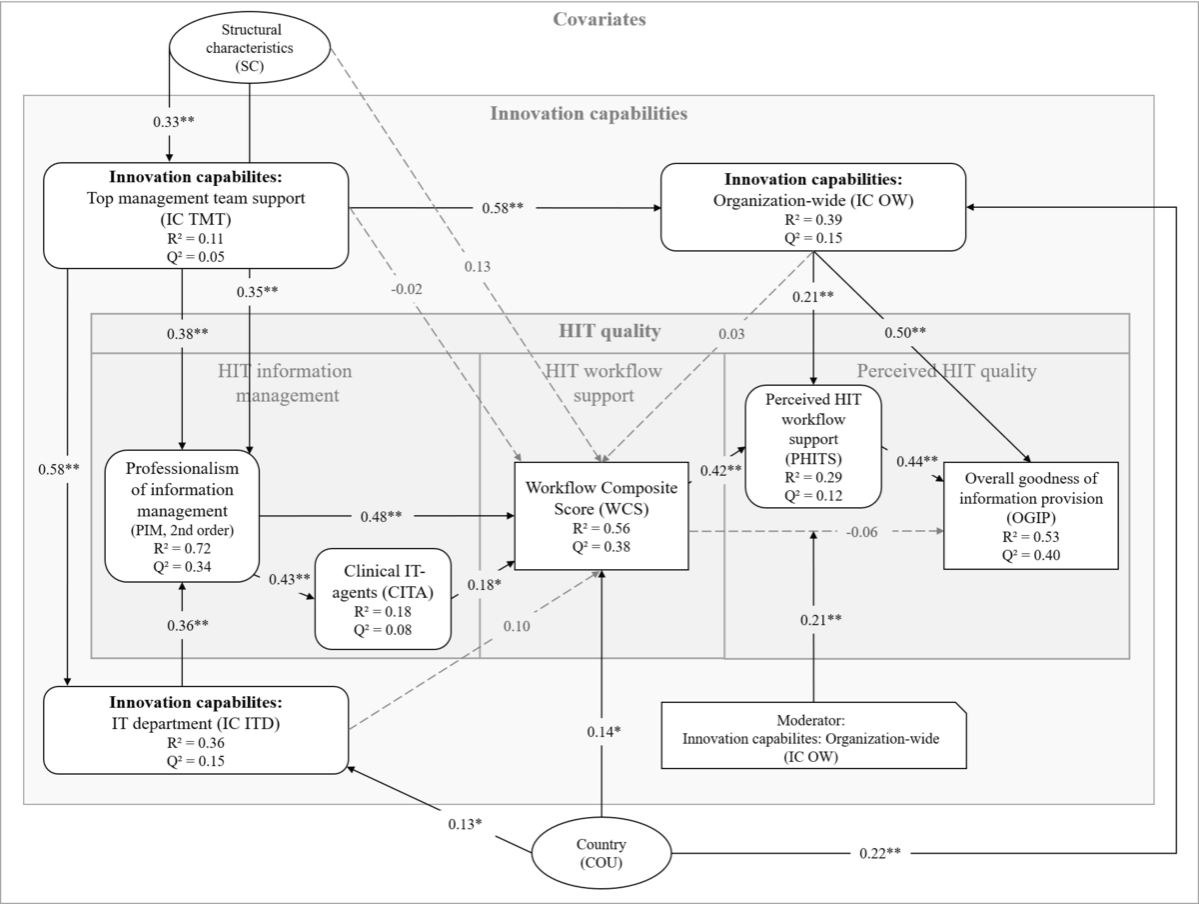
The structural model of innovation and quality of health information technology with path coefficients, explained variance (R²), and predictive relevance measures (Q²) of the endogenous constructs. Latent constructs are displayed with rounded edges, the exogenous covariates as ellipses and the moderator variable with a cut-off corner. *P<.05; **P<.01. HIT: health information technology.

The 21 hypotheses (Table 2) led to a variety of interrelationships in the structural model in terms of direct, mediated, and moderated effects. Approximately 50% of the variance in the key constructs for measuring HIT quality, the HIT workflow support (as measured by the WCS), and the perceived overall goodness of information provision (OGIP) could be explained by the model (Figure 2).

Within the HIT quality domain, the results showed a strong effect of PIM on the WCS with a path coefficient estimate of 0.48 (*P*<.001). This association was partially mediated by the use of clinical IT agents to a small but significant extent (Multimedia Appendix 7). Furthermore, WCS was associated with OGIP via an indirect effect between the 2, which was mediated by the perceived HIT workflow support. The exact *P* values of the path coefficients are shown in Multimedia Appendix 8.

Within the innovation layer, the IC TMT exhibited a strong effect on IC ITD and IC OW.

Furthermore, the model revealed a strong association between innovation and quality at various levels (the total and indirect effects are given in Multimedia Appendix 7): the ICs of the TMT and of the IT department significantly and similarly affected PIM, whereas IC OW had a strong effect on the perceived HIT quality in terms of OGIP and a weaker but still significant effect related to perceived HIT workflow support. Contrary to some of our initial assumptions, as expressed in hypotheses H9, H10, and H12, there was no significant direct effect of any of the constructs representing IC on the WCS (Table 4). Instead, the results showed significant indirect effects of IC TMT and IC ITD on the WCS mediated by PIM (Multimedia Appendix 7). The effect of the WCS on OGIP, which did not become significant, was, however, significantly moderated by IC OW (hypothesis H15). In summary, ICs possessed many points of application at the HIT quality path, that is, at the beginning influencing PIM and later affecting the overall quality of information provision for patient care.

**Table 4:** Summarized results of the hypothesis tests in reference to *P* values <.05.

Hypothesis	Support by the model
H1: PIM^a^ has a positive effect on the WCS^b^	Supported
H2: The effect of PIM on the WCS is partly mediated by clinical IT^c^ agents	Supported
H3: The WCS has a positive effect on perceived HIT^d^ workflow support	Supported
H4: The WCS has a positive effect on the overall goodness of information provision	Not supported
H5: Perceived HIT workflow support has a positive effect on the overall goodness of information provision	Supported
H6: Innovation capabilities: Top management team support has a positive effect on organization-wide innovation capability	Supported
H7: Innovation capabilities: Top management team support has a positive effect on the innovation capability of the IT department	Supported
H8: Innovation capabilities: Top management team support has a positive effect on PIM	Supported
H9: Innovation capabilities: Top management team support has a positive effect on the WCS	Not supported
H10: Innovation capability of the IT department has a positive effect on the WCS	Not supported
H11: Innovation capability of the IT department has a positive effect on PIM	Supported
H12: Organization-wide innovation capability has a positive effect on the WCS	Not supported
H13: Organization-wide innovation capability has a positive effect on perceived HIT workflow support	Supported
H14: Organization-wide innovation capability has a positive effect on the overall goodness of information provision	Supported
H15: Organization-wide innovation capability positively moderates the relationship between the WCS and the overall goodness of information provision	Supported
H16: Structural characteristics have a positive effect on the WCS	Not supported
H17: Structural characteristics have a positive effect on PIM	Supported
H18: Structural characteristics have a positive effect on innovation capabilities: top management team support	Supported
H19: Country has a positive effect on the WCS	Supported
H20: Country has a positive effect on the organization-wide innovation capability	Supported
H21: Country has a positive effect on the innovation capability of the IT department	Supported

^a^PIM: professionalism of information management.

^b^WCS: Workflow Composite Score.

^c^IT: information technology.

^d^HIT: health information technology.

With regard to the covariates, the country had a significant effect on the WCS and was also associated with higher degrees of IC ITD and IC TMT, albeit with rather small effect sizes f² (Multimedia Appendix 8). The organization’s structural characteristics did not exhibit a direct effect on the WCS in our model but instead on the *preceding* latent variables in the model, namely, PIM and IC TMT.

## Discussion

### Principal Findings

On the basis of data from 232 hospitals in Austria, Germany, and Switzerland, a sociotechnical IQ_HIT_ model was developed and tested. To the best of our knowledge, this is the first model that investigates HIT quality in light of the organizations’ ability to innovate. It does so in a strictly empirical manner using a validated instrument. The model sets out the internal composition of HIT quality in establishing a consecutive connection between HIT information management, HIT workflow support, and perceived quality. Furthermore, an organization’s ICs were positively associated with HIT quality at various levels. Most notably, an innovation-friendly attitude on the TMT level appeared to strongly but indirectly facilitate HIT-based workflow support, mediated by professional information management practices.

### The Inner Workings of HIT Quality

At the core of the IQ_HIT_ model, the WCS was used to measure HIT quality in terms of the workflow to support the IT solutions provided for improving patient care. The WCS is a multifaceted indicator that consists of a plethora of underlying items (Multimedia Appendix 2). By incorporating it, the model considers the complexity of interdepartmental and multifunctional health information systems.

According to the model, HIT workflow support depends on professional information management, that is, professionally conceptualized and performed activities at the strategic, tactical, and operational levels, as has been conjectured by Winter et al [34] and empirically conceptualized by Thye et al [36]. Only the HIT workflow support that is managed in an orderly and professional manner by the IT department can work properly regarding data and information provision, IT functions in place, their integration with one another, and the ability to distribute the data and the information to the point of care. Part of this effect is mediated by the presence of clinical IT agents, confirming the importance of establishing a formal link between IT department information management and clinical end users. Interestingly, the structural characteristics (bed count and teaching status) did not affect the HIT workflow support directly but only via the mediating effect of professional information management. This is rather surprising, as most studies suggest a direct link, particularly between the size of an organization and its HIT use [25].

HIT quality was conceptualized to encompass both, a technical component that bundles manifest, self-reported attributes about the information system, that is, the WCS, and a subjective judgment about its perceived quality. According to the CIOs’ viewpoint, the very abstract judgment of the perceived goodness of information provision appears to not be directly linked to the WCS but requires some intermediate interpretation, that is, the perceived HIT workflow support, which refers to a more detailed perspective of admission, ward rounds, presurgery and postsurgery, and discharge processes. This also suggests that there is no strict automatism between a high degree of HIT quality in terms of its technical components and the perceived quality of information provision in an organization. This points to the need for good implementation practices of HIT interventions to successfully reap their benefits.

### Innovation Capabilities in Health Care Organizations

The IQ_HIT_ model also specifies the inner fabric of organizational IC. The underlying scales yielded good psychometric properties and reflected an innovation-friendly attitude and behavior at different organizational levels: at the executive level (IC TMT), the items mirror the motivational and monetary support of the TMT for IT innovation and their proactive engagement with respective projects as part of the organization’s vision. Similar to the views in the Upper Echelons Theory, which stresses the crucial role of senior leadership in fostering innovation, this factor had a strong predictive relevance across the model [94]. IC ITD reflect the kind of CIO leadership that facilitates creativity, communication, and participation of end users. On the third level (IC OW), openness and widespread flexibility for embracing new IT solutions that prevail throughout the organization at large were the defining elements. Most of these characteristics were suspected [47,95,96] and partly known [27,97,98] to facilitate innovation in a variety of contexts; however, the way they statistically cluster along different organizational levels and their different effects has not been specified before. Therefore, the innovative capacity of health organizations cannot be viewed as monolithic blocks or mere buzzwords. Its contents are woven throughout various organizational levels to varying degrees.

This study did not explicitly focus on how these capabilities can be built or how they are determined. However, when controlling for the covariates, we found that TMT support is a function of certain structural characteristics, namely, a higher bed count and teaching status, both of which can be interpreted as indicators of greater financial flexibility in terms of slack resources. However, ICs at the IT department and the organization at large depend on the respective country. More precisely, these 2 domains are more pronounced in Austria and Switzerland than in Germany, which corresponds well with previous findings on different samples [11,49].

### HIT Implementation Between Innovation and Quality

Traditionally, empirical research conducted on HIT quality has frequently disregarded aspects of innovation, and both have often been discussed separately from one another [75]. Our model establishes a connection between the two by showing that attaining high levels of HIT quality is facilitated and mediated by an organization’s ability to create space for creativity, agility, and communication in relation to IT-based innovation.

**Figure 3:**
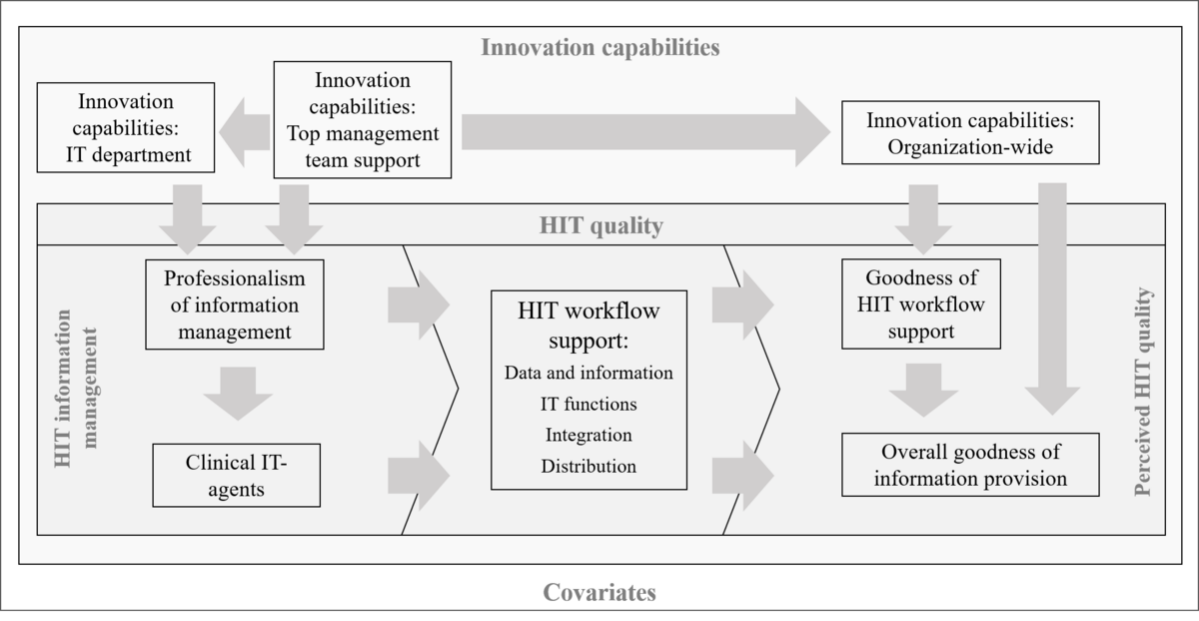
The innovation and quality of health information technology model. HIT: health information technology; IT: information technology.

Overall, the structural model (Figure 2) can be translated into a more schematic model (Figure 3) based on the major findings. It shows that PIM mediates the effects of the 2 IC domains—IC TMT and IC ITD—on HIT workflow support, which illustrates the interplay between the *right attitude* toward innovation and formalized management practices for innovational strength. The attitude and intent to innovate play an important role in and of itself; however, professional information management is needed for the practical execution of this intention to improve HIT workflow support.

Furthermore, we found IC OW to partly moderate the relationship between the HIT workflow support and the perceived OGIP, implying that there might actually be a direct effect between the 2 as long as the organization is agile, flexible, and open toward IT (equals high levels of IC OW). This could be interpreted as an indication that an organization-wide positive attitude toward using the IT in place, irrespective of how advanced it actually is, leads to better information provision in the clinical care processes, at least from the vantage point of CIOs. Overall, it becomes clear that ICs are not only needed at the TMT level but also at the IT department level and throughout the organization to establish high-quality HIT solutions. Executive managers and policy makers should therefore consider how to establish higher levels of these capabilities at various levels.

### Limitations

Our study had several limitations. Most notably, this is an observational study, and despite the statistical specifications that might suggest otherwise, it cannot be inferred that the relationship between constructs is truly causal. For instance, there might be temporal displacements between the current beliefs of executives and higher degrees of HIT quality as implementation processes take time [99].

Furthermore, this sociotechnical model reflects the perspective of the CIOs and their points of view of the HIT cosmos and ICs. This is both a strength and a weakness. The strength is its consistency and authenticity regarding technical and organizational issues related to IT. Its weakness is the CIOs cannot accurately evaluate clinicians’ view on the timely and correct provision of data and information (ie, the *right side* of the model), which requires a more detailed assessment in future research. Ultimately, the clinical outcome is the improvement or stabilization of the patient’s condition. None of this is captured in this model, as it mirrors the vantage point of CIOs. The next step will be to develop a model that incorporates the views of physicians and nurses. This approach can also cope with potential common-method biases. The sample is also based on voluntary participation, which is why we cannot rule out a nonresponse bias in the data.

Finally, not all possibly relevant factors at play can be accurately accounted for in one model, which is reflected by the R² values that leave parts of the variance in the endogenous constructs unexplained. Given these limitations, further studies are needed to validate and differentiate the relationships between and within IC and HIT quality, and our model provides various access points to do so.

### Conclusions

On the basis of survey data provided by the CIOs of 232 hospitals, we proposed a sociotechnical IQ_HIT_ model to explain how organizational innovation relates to various facets of HIT quality. Although some associations in the model could be presumed by the literature, it clearly and uniquely highlights the key role of ICs and information management for HIT-based workflow support. Thus, it demonstrates that innovation and quality do not contradict each other. In particular, an innovation-friendly attitude of TMT and the IT department determines the degree of HIT workflow support, albeit not directly, but by means of professional information management practices that eventually facilitate the perceived goodness of information provision for patient care. This suggests that managers of health organizations should strive for both a more pronounced culture toward innovation and professional information management to translate such a culture into HIT quality. Furthermore, the IQ_HIT_ model might be useful for studies on HIT adoption and diffusion and for the definition of HIT maturity models. To this end, it provides validated measurement scales that can be utilized in future research.

## Appendix

Multimedia Appendix 1 Measurement models and underlying items (questionnaire part A).

Multimedia Appendix 2 Workflow Composite Score (WCS): structure and underlying items (questionnaire part B).

Multimedia Appendix 3 Descriptive statistics of the Workflow Composite Score (WCS; n=232).

Multimedia Appendix 4 Convergent validity and internal consistency of the measurement models with bias corrected 95% CIs.

Multimedia Appendix 5 Convergent validity and internal consistency of lower order constructs reflecting the latent variable “Professionalism of Information Management (PIM)” with bias corrected 95% CIs.

Multimedia Appendix 6 Heterotrait-Monotrait (HTMT) ratios.

Multimedia Appendix 7 Total effects and total indirect effects of the structural model with bias corrected 95% CIs and significance tests of the path coefficients.

Multimedia Appendix 8 Direct path coefficients with bias corrected 95% CIs, significance tests of path coefficients, and their effect sizes.

## Abbreviations

CIO: chief information officer
HIT: health information technology
IT: information technology
IC: innovation capability
IC ITD: innovation capabilities at the information technology department level
IC TMT: innovation capabilities at the top management team level
IC OW: innovation capabilities at the organization-wide level
IQHIT: innovation and quality of HIT
OGIP: overall goodness of information provision
PIM: professionalism of information management
TMT: top management team
WCS: Workflow Composite Score
