# Supplementary material for: The Effect of Innovation Capabilities of Health Care Organizations on the Quality of Health Information Technology: Model Development With Cross-sectional Data
Source: JMIR Med Inform. 2021 Mar 15;9(3):e23306. doi: 10.2196/23306 (PMC8077601; doi:10.2196/23306)
Supplement: Multimedia Appendix 1 [file medinform_v9i3e23306_app1.docx]

**Multimedia Appendix 1. Measurement models and underlying items (questionnaire part A).**

| Measurement Model | | Value, mean (SD)^a^ | Indicator | | | |
| --- | --- | --- | --- | --- | --- | --- |
|  |  |  | Question | Code | Label/Sub-question | Scale/Categories |
| Professionalism of Information Management  (PIM)^b^ | Strategic Information Management (PIM_1) | 54.2  (24.6) | Are the following IT management activities or procedures performed in your hospital? | PIM_1_S1 | Preparation and further development of an information management strategy | Categorical (not at all, irregularly, regularly) |
|  |  |  |  | PIM_1_S2 | Strategic controlling in terms of IT project management including project portfolios |  |
|  |  |  |  | PIM_1_S3 | Long-term finance and investment planning |  |
|  |  |  |  | PIM_1_S4 | Strategic risk management (e.g. maintenance of emergency plans) |  |
|  |  |  |  | PIM_1_S5 | Evaluation of IT efficiency benefits |  |
|  |  |  |  | PIM_1_S6 | Evaluation of user satisfaction |  |
|  | Tactical Information Management (PIM_2) | 60.7  (17.4) | Are the following IT management activities or procedures performed in your hospital? | PIM_2_T1 | Workflow analysis and evaluation (e.g. process modeling, evaluation of the current state) | Categorical (not at all, irregularly, regularly) |
|  |  |  |  | PIM_2_T2 | System specification (e.g. requirements definition, specifications, migration plan) |  |
|  |  |  |  | PIM_2_T3 | System selection (e.g. market analysis, tendering, bid comparison) |  |
|  |  |  |  | PIM_2_T4 | System implementation (e.g. implementation strategy and adaptation) |  |
|  |  |  |  | PIM_2_T5 | Further cooperation with manufacturers (for product development/enhancement) |  |
|  | Operational Information Management (PIM_3) | 85.7  (20.1) | Are the following IT management activities or procedures performed in your hospital? | PIM_3_O1 | Application management and maintenance | Categorical (not at all, irregularly, regularly) |
|  |  |  |  | PIM_3_O2 | Management and monitoring of the technical performance (infrastructure and networks) |  |
|  |  |  |  | PIM_3_O3 | Training of clinical end users |  |
|  |  |  |  | PIM_3_O4 | Continuous operation of the help desk/service desk |  |
| Innovation Capability: Top Management Team Support  (IC TMT) | | 62.6  (19.9) | Please indicate your (dis-) agreement with the following statements. | IC_TMT_1 | “Our hospital has a well-defined future vision that is shared by the IT department.” | Five-point Likert scale (strongly disagree-strongly agree). |
|  |  |  |  | IC_TMT_2 | “Our executive board regularly seeks the exchange with the CIO.” |  |
|  |  |  |  | IC_TMT_3 | “I have often been given positive feedback from the executive board for contributing innovative ideas.” |  |
|  |  |  |  | IC_TMT_4 | "Our executive board explicitly calls for proposals of innovative eHealth solutions." |  |
|  |  |  |  | IC_TMT_5 | “Our executive board actively promotes the initiation of new IT projects." |  |
|  |  |  |  | IC_TMT_6 | “Our executive board regularly perceives IT as a mere expense factor.” (reverse coded) |  |
| Innovation Capability of the IT Department (IC ITD) | | 71.1  (15.5) | Please indicate your (dis-) agreement with the following statements. | IC_ITD_1 | “In the IT department, we regularly discuss new IT solutions with representatives of the specialist departments (clinical users).” | Five-point Likert scale (strongly disagree-strongly agree). |
|  |  |  |  | IC_ITD_2 | “In our team, creative ideas and suggestions for new IT applications are carefully listened to and discussed.” |  |
|  |  |  |  | IC_ITD_3 | “Everyone in my team needs certain degrees of freedom in order to come up with the best possible solutions.” |  |
|  |  |  |  | IC_ITD_4 | “Employee creativity is a major driving force in our IT department.” |  |
|  |  |  |  | IC_ITD_5 | “Our IT team has often shown a strong sense of cohesion.” |  |
| Organization-Wide Innovation Capability (IC OW) | | 59.4  (17.7) | Please indicate your (dis-) agreement with the following statements. | IC_OW_1 | “Our entire hospital shows great agility and flexibility when it comes to implementing and using new IT solutions.” | Five-point Likert scale (strongly disagree-strongly agree). |
|  |  |  |  | IC_OW_2 | “In our hospital, new IT projects are openly communicated and discussed between all participants.” |  |
|  |  |  |  | IC_OW_3 | “Our hospital is far too inflexible at all levels of hierarchy to use IT solutions in a meaningful way.” (reverse coded) |  |
|  |  |  |  | IC_OW_4 | “The responsiveness of our IT landscape to new requirements is excellent.” |  |
|  |  |  |  | IC_OW_5 | “Our users and employees often have a fundamental aversion to IT.” (reverse coded) |  |
| Perceived HIT Workflow Support (PHITS) | | 69.8  (15.7) | How well do the various IT-systems support the following workflows overall? | PHITS _1 | Admission | Grades (very good, good, satisfactory, sufficient, poor) |
|  |  |  |  | PHITS _2 | Ward rounds |  |
|  |  |  |  | PHITS _3 | Pre-surgery |  |
|  |  |  |  | PHITS _4 | Post-surgery |  |
|  |  |  |  | PHITS _5 | Discharge |  |
| Clinical IT-Agents (CITA) | | 31.5  (30.9) | Are there any physicians or nurses in your hospital who are officially responsible for IT matters? | CITA_1 | At least one physician is officially responsible for IT matters. | Binary (Yes/No) |
|  |  |  |  | CITA_2 | At least one nurse is officially responsible for IT matters. |  |
| Overall Goodness of Information Provision^c^ | | 68.4  (19.3) | Please indicate your (dis-) agreement with the following statement. | OGIP | Our hospital always provides the right information, at the right time, at the right place, for the right persons, and in the right quality to support clinical processes. | Five-point Likert scale (strongly disagree-strongly agree). |
| Structural Characteristics (SC)^d^ | |  | How many beds does your hospital have? | SC_1 |  | Metric (bed count) |
|  |  |  | What is the teaching status of your hospital? | SC_2 |  | Categorical (nonteaching, minor teaching, major teaching) |
| Country^cde^ | |  |  | COU |  | Categorical (Austria, Germany, Switzerland) |
| Workflow Composite Score (WCS)^cf^ | | 55.9  (13.7) |  | WCS |  | Metric (ranges from 0 to 100 points) |

^a^ Sum based composite scores for each measurement model, transformed to range from 0-100.

^b^ This higher order construct PIM has a mean (SD) of 64.8 (19.2).

^c^ Single item scale.

^d^ See Table 3 for descriptive information.

^e^ No question associated with this variable, as it was not part of the questionnaire, but was added subsequently.

^f^ Calculated based on a fixed structure of 146 underlying questionnaire items (questionnaire part B, see Multimedia Appendix 2).
