# Supplementary material for: The Effect of Innovation Capabilities of Health Care Organizations on the Quality of Health Information Technology: Model Development With Cross-sectional Data
Source: JMIR Med Inform. 2021 Mar 15;9(3):e23306. doi: 10.2196/23306 (PMC8077601; doi:10.2196/23306)
Supplement: Multimedia Appendix 3 [file medinform_v9i3e23306_app3.docx]

## **Multimedia Appendix 3. Descriptive statistics of the Workflow Composite Score (WCS) (n=232).**

| Score | | Mean (SD) | Min. | Max. |
| --- | --- | --- | --- | --- |
|  | |  |  |  |
| Workflow Composite Score | | 55.9 (13.7) | 21.9 | 83.3 |
| **Sub-scores** | |  |  |  |
|  | Admission | 45.3 (15.2) | 14.2 | 83.1 |
|  | Ward round | 57.8 (17.2) | 19.4 | 88.4 |
|  | Pre-surgery | 64.2 (12.0) | 23.5 | 86.2 |
|  | Post-surgery | 61.8 (13.3) | 20.7 | 86.6 |
|  | Discharge | 56.5 (12.7) | 22.7 | 88.3 |
