# Supplementary material for: The Effect of Innovation Capabilities of Health Care Organizations on the Quality of Health Information Technology: Model Development With Cross-sectional Data
Source: JMIR Med Inform. 2021 Mar 15;9(3):e23306. doi: 10.2196/23306 (PMC8077601; doi:10.2196/23306)
Supplement: Multimedia Appendix 4 [file medinform_v9i3e23306_app4.docx]

## **Multimedia Appendix 4. Convergent validity and internal consistency of the measurement models with bias corrected 95% confidence intervals (CI). Please refer to Multimedia Appendix 1 for the indicator labels.**

| Latent Variable | Indicator | Outer Loading / Weights ^a^ [95% CI] | Composite Reliability (CR)^a^ [95% CI] | Cronbach’s α^a^ [95% CI] | Average Variance Extracted (AVE)^a^ [95% CI] |
| --- | --- | --- | --- | --- | --- |
|  |  |  |  |  |  |
| Professionalism of Information Management (PIM)^b^ | PIM_1 | .78 [.69, .85] | .76 [.69, .81] | .75 [.67, .81] | .51 [.43, .58] |
|  | PIM_2 | .74 [.65, .81] |  |  |  |
|  | PIM_3 | .62 [.47, .73] |  |  |  |
| Innovation Capability: Top Management Team Support (IC TMT) | IC_TMT_1 | .80 [.70, .89] | .86 [.82, .89] | .86 [.82, .89] | .52 [.44, .58] |
|  | IC_TMT_2 | .75 [.61, .85] |  |  |  |
|  | IC_TMT_3 | .74 [.60, .86] |  |  |  |
|  | IC_TMT_4 | .72 [.59, .82] |  |  |  |
|  | IC_TMT_5 | .67 [.55, .78] |  |  |  |
|  | IC_TMT_6 | .57 [.41, .69] |  |  |  |
| Innovation Capability of the IT Department  (IC ITD) | IC_ITD_1 | .87 [.77, .98] | .83 [.78, .87] | .84 [.79, .87] | .51 [.42, .58] |
|  | IC_ITD_2 | .82 [.73, .92] |  |  |  |
|  | IC_ITD_3 | .64 [.45, .76] |  |  |  |
|  | IC_ITD_4 | .61 [.45, .71] |  |  |  |
|  | IC_ITD_5 | .58 [.41, .71] |  |  |  |
| Organization-Wide Innovation Capability  (IC OW) | IC_OW_1 | .82 [.73, .92] | .81 [.75, .85] | .81 [.76, .85] | .46 [.39, .53] |
|  | IC_OW_2 | .71 [.57, .81] |  |  |  |
|  | IC_OW_3 | .66 [.51, .77] |  |  |  |
|  | IC_OW_4 | .62 [.50, .73] |  |  |  |
|  | IC_OW_5 | .57 [.42, .67] |  |  |  |
| Perceived HIT Workflow Support  (PHITS) | PHITS_1 | .80 [.72, .86] | .89 [.85, .91] | .89 [.85, .91] | .61 [.53, .67] |
|  | PHITS_2 | .80 [.70, .86] |  |  |  |
|  | PHITS_3 | .78 [.73, .83] |  |  |  |
|  | PHITS_4 | .76 [.67, .83] |  |  |  |
|  | GITPC_5 | .76 [.66, .82] |  |  |  |
| Clinical IT-Agents  (CITA) | CITA_1 | .85 [.73, .96] | .75 [.65, .82] | .74 [.65, .82] | .60 [.48, .70] |
|  | CITA_2 | .70 [.55, .82] |  |  |  |
| Structural Characteristics (SC)^c^ | ISC_1 | .60 [.36, .80] |  |  |  |
|  | ISC_2 | .56 [.31, .77] |  |  |  |

^a^ Common acceptance ranges: Outer Loadings / Weights at least > .40, recommended >.70; CR & Cronbach’s α between .70 and .90; AVE > .50.

^b^ Second order construct, see Multimedia Appendix 5 for the underlying indicators.

^c^ Formative measurement model.
