# Supplementary material for: The Effect of Innovation Capabilities of Health Care Organizations on the Quality of Health Information Technology: Model Development With Cross-sectional Data
Source: JMIR Med Inform. 2021 Mar 15;9(3):e23306. doi: 10.2196/23306 (PMC8077601; doi:10.2196/23306)
Supplement: Multimedia Appendix 5 [file medinform_v9i3e23306_app5.docx]

## **Multimedia Appendix 5. Convergent validity and internal consistency of lower order constructs reflecting the latent variable “Professionalism of Information Management (PIM)” with bias corrected 95% confidence intervals (CI).**

| Lower Order Constructs | Indicator | Outer Loading^a^ [95% CI] | Composite Reliability (CR)^a^ [95% CI] | Cronbach’s α^a^ [95% CI] | Average Variance Extracted (AVE)^a^ [95% CI] | Loading^a^ with Higher Order Construct PIM [95% CI] |
| --- | --- | --- | --- | --- | --- | --- |
| Strategic Information Management (PIM_1) | PIM_1_S1 | .84 [.80, .87] | .87 [.85, .89] | .82 [.78, .85] | .53 [.49, .58] | .88 [.84, .91] |
|  | PIM_1_S2 | .83 [.78, .87] |  |  |  |  |
|  | PIM_1_S3 | .67 [.56, .75] |  |  |  |  |
|  | PIM_1_S4 | .77 [.71, .83] |  |  |  |  |
|  | PIM_1_S5 | .63 [.54, .71] |  |  |  |  |
|  | PIM_1_S6 | .60 [.49, .68] |  |  |  |  |
| Tactical Information Management (PIM_2) | PIM_2_T1 | .76 [.70, .80] | .87 [.85, .90] | .82 [.77, .86] | .58 [.53, .63] | .87 [.82, .90] |
|  | PIM_2_T2 | .81 [.76, .85] |  |  |  |  |
|  | PIM_2_T3 | .80 [.74, .85] |  |  |  |  |
|  | PIM_2_T4 | .77 [.70, .82] |  |  |  |  |
|  | PIM_2_T5 | .67 [.59, .74] |  |  |  |  |
| Operational Information Management (PIM_3) | PIM_3_O1 | .83 [.75, .88] | .83 [.78, .87] | .73 [.63, .81] | .56 [.48, .63] | .69 [.56, .77] |
|  | PIM_3_O2 | .77 [.64, .86] |  |  |  |  |
|  | PIM_3_O3 | .65 [.55, .74] |  |  |  |  |
|  | PIM_3_O4 | .72 [.60, .80] |  |  |  |  |

^a^ Common acceptance ranges: Loadings at least > .40, recommended >.70; CR & Cronbach’s α between .70 and .90; AVE > .50.
