# Supplementary material for: The Effect of Innovation Capabilities of Health Care Organizations on the Quality of Health Information Technology: Model Development With Cross-sectional Data
Source: JMIR Med Inform. 2021 Mar 15;9(3):e23306. doi: 10.2196/23306 (PMC8077601; doi:10.2196/23306)
Supplement: Multimedia Appendix 6 [file medinform_v9i3e23306_app6.docx]

## **Multimedia Appendix 6. HTMT ratios.**

|  | OGIP | IC ITD | IC TMT | IC OW | CITA | PIM | WCS | PHITS |
| --- | --- | --- | --- | --- | --- | --- | --- | --- |
| IC ITD | .33  [.17, .47] |  |  |  |  |  |  |  |
| IC TMT | .36  [.22, .48] | .56  [.42, .68] |  |  |  |  |  |  |
| IC OW | .62  [.51, .70] | .49  [.35, .62] | .57  [.43, .70] |  |  |  |  |  |
| CITA | .12  [.02, .26] | .29  [.17, .41] | .39  [.24, .53] | .31  [.18, .44] |  |  |  |  |
| PIM | .25  [.11, .39] | .69  [.54, .80] | .70  [.60, .80] | .48  [.34, .62] | .43  [.29, .57] |  |  |  |
| WCS | .33  [.20, .44] | .55  [.44, .64] | .51  [.40, .61] | .38  [.24, .51] | .45  [.31, .57] | .72  [.61, .81] |  |  |
| PHITS | .54  [.41, .64] | .31  [.17, .44] | .40  [.24, .54] | .36  [.23, .49] | .10  [.03, .16] | .33  [.19, .47] | .49  [.37, .59] |  |
| COU | .19  [.08, .29] | .15  [.04, .30] | .06  [.01, .08] | .24  [.12, .36] | .10  [.02, .25] | .04  [.01, .06] | .17  [.06, .30] | .13  [.05, .22] |
