# Supplementary material for: The Effect of Innovation Capabilities of Health Care Organizations on the Quality of Health Information Technology: Model Development With Cross-sectional Data
Source: JMIR Med Inform. 2021 Mar 15;9(3):e23306. doi: 10.2196/23306 (PMC8077601; doi:10.2196/23306)
Supplement: Multimedia Appendix 7 [file medinform_v9i3e23306_app7.docx]

## **Multimedia Appendix 7. Total effects and total indirect effects of the structural model with bias corrected 95% confidence intervals (CI) and significance tests of the path coefficients.**

| Path | | Coefficient  [95% CI] | *P* Value |
| --- | --- | --- | --- |
| **Total Effects** | |  |  |
|  | Innovation Capability of the IT Department (IC ITD) 🡪 Overall Goodness of Information Provision (OGIP) | .03 [-.01, .09] | .18 |
|  | Innovation Capability of the IT Department (IC ITD) 🡪 Clinical IT-Agents (CITA) | .15 [.08, .25] | <.001 |
|  | Innovation Capability of the IT Department (IC ITD) 🡪 Workflow Composite Score (WCS) | .31 [.15, .46] | <.001 |
|  | Innovation Capability of the IT Department (IC ITD) 🡪 Perceived HIT Workflow Support (PHITS) | .13 [.06, .21] | .001 |
|  | Innovation Capability: Top Management Team Support (IC TMT) 🡪 Overall Goodness of Information Provision (OGIP) | .38 [.27, .48] | <.001 |
|  | Innovation Capability: Top Management Team Support (IC TMT) 🡪 Clinical IT-Agents (CITA) | .25 [.16, .36] | <.001 |
|  | Innovation Capability: Top Management Team Support (IC TMT) 🡪 Professionalism of Information Management (PIM) | .59 [.47, .70] | <.001 |
|  | Innovation Capability: Top Management Team Support (IC TMT) 🡪 Workflow Composite Score (WCS) | .38 [.27, .50] | <.001 |
|  | Innovation Capability: Top Management Team Support (IC TMT) 🡪 Perceived HIT Workflow Support (PHITS) | .30 [.21, .40] | <.001 |
|  | Organization-Wide Innovation Capability (IC OW) 🡪 Overall Goodness of Information Provision (OGIP) | .59 [.46, .71] | <.001 |
|  | Organization-Wide Innovation Capability (IC OW) 🡪 Perceived HIT Workflow Support (PHITS) | .22 [.07, .37] | .01 |
|  | Clinical IT-Agents (CITA) 🡪 Overall Goodness of Information Provision (OGIP) | .02 [.00, .06] | .23 |
|  | Clinical IT-Agents (CITA) 🡪 Perceived HIT Workflow Support (PHITS) | .07 [.01, .15] | .03 |
|  | Professionalism of Information Management (PIM) 🡪 Overall Goodness of Information Provision (OGIP) | .06 [-.02, .17] | .17 |
|  | Professionalism of Information Management (PIM) 🡪 Workflow Composite Score (WCS) | .55 [.36, .74] | <.001 |
|  | Professionalism of Information Management (PIM) 🡪 Perceived HIT Workflow Support (PHITS) | .23 [.10, .42] | .01 |
|  | Structural Characteristics (SC) 🡪 Overall Goodness of Information Provision (OGIP) | .16 [.09, .23] | <.001 |
|  | Structural Characteristics (SC) 🡪 Innovation Capability of the IT Department (IC ITD) | .19 [.11, .27] | <.001 |
|  | Structural Characteristics (SC) 🡪 Organization-Wide Innovation Capability (IC OW) | .19 [.12, .27] | <.001 |
|  | Structural Characteristics (SC) 🡪 Clinical IT-Agents (CITA) | .23 [.15, .33] | <.001 |
|  | Structural Characteristics (SC) 🡪 Professionalism of Information Management (PIM) | .55 [.44, .65] | <.001 |
|  | Structural Characteristics (SC) 🡪 Workflow Composite Score (WCS) | .46 [.34, .56] | <.001 |
|  | Structural Characteristics (SC) 🡪 Perceived HIT Workflow Support (PHITS) | .23 [.17, .31] | <.001 |
|  | Workflow Composite Score (WCS) 🡪 Overall Goodness of Information Provision (OGIP) | .10 [.00, .23] | .14 |
|  | Country (COU) 🡪 Overall Goodness of Information Provision (OGIP) | .15 [.08, .22] | <.001 |
|  | Country (COU) 🡪 Clinical IT-Agents (CITA) | .02 [.00, .05] | .07 |
|  | Country (COU) 🡪 Professionalism of Information Management (PIM) | .05 [.01, .10] | .06 |
|  | Country (COU) 🡪 Workflow Composite Score (WCS) | .17 [.03, .31] | .01 |
|  | Country (COU) 🡪 Perceived HIT Workflow Support (PHITS) | .12 [.06, .18] | <.001 |
| **Total indirect effects** | |  |  |
|  | Innovation Capability: Top Management Team Support (IC TMT) 🡪 Professionalism of Information Management (PIM) | .21 [.11, .33] | <.001 |
|  | Innovation Capability: Top Management Team Support (IC TMT) 🡪 Workflow Composite Score (WCS) | .41 [.25, .62] | <.001 |
|  | Innovation Capability of the IT Department (IC ITD) 🡪 Workflow Composite Score (WCS) | .20 [.08, .41] | .03 |
|  | Organization-Wide Innovation Capability (IC OW) 🡪 Overall Goodness of Information Provision (OGIP) | .10 [.04, .18] | .01 |
|  | Organization-Wide Innovation Capability (IC OW) 🡪 Perceived HIT Workflow Support (PHITS) | .01 [-.07, .90] | .78 |
|  | Professionalism of Information Management (PIM) 🡪 Workflow Composite Score (WCS) | .08 [.02, .16] | .04 |
|  | Structural Characteristics (SC) 🡪 Professionalism of Information Management (PIM) | .20 [.12, .28] | <.001 |
|  | Structural Characteristics (SC) 🡪 Workflow Composite Score (WCS) | .32 [.19, .49] | <.001 |
|  | Workflow Composite Score (WCS) 🡪 Overall Goodness of Information Provision (OGIP) | .18 [.11, .29] | <.001 |
|  | Country (COU) 🡪 Workflow Composite Score (WCS) | .05 [-.01, .10] | .10 |
