# Supplementary material for: The Effect of Innovation Capabilities of Health Care Organizations on the Quality of Health Information Technology: Model Development With Cross-sectional Data
Source: JMIR Med Inform. 2021 Mar 15;9(3):e23306. doi: 10.2196/23306 (PMC8077601; doi:10.2196/23306)
Supplement: Multimedia Appendix 8 [file medinform_v9i3e23306_app8.docx]

## **Multimedia Appendix 8. Direct path coefficients with bias corrected 95% confidence intervals (CI), significance tests of path coefficients and their effect sizes.**

| Path | Coefficient [95% CI] | *P* Values | f² |
| --- | --- | --- | --- |
| Innovation Capability of the IT Department (IC ITD) 🡪 Professionalism of Information Management (PIM) | .36 [.20, .51] | <.001 | .31 |
| Innovation Capability of the IT Department (IC ITD) 🡪 Workflow Composite Score (WCS) | .10 [-.14, .31] | .37 | .01 |
| Innovation Capability: Top Management Team Support (IC TMT) 🡪 Innovation Capability of the IT Department (IC ITD) | .58 [.43, .70] | <.001 | .53 |
| Innovation Capability: Top Management Team Support (IC TMT) 🡪 Organization-Wide Innovation Capability (IC OW) | .58 [.44, .70] | <.001 | .54 |
| Innovation Capability: Top Management Team Support (IC TMT) 🡪 Professionalism of Information Management (PIM) | .38 [.23, .53] | <.001 | .33 |
| Innovation Capability: Top Management Team Support (IC TMT) 🡪 Workflow Composite Score (WCS) | -.02 [-.26, .17] | .84 | .01 |
| Organization-Wide Innovation Capability (IC OW) 🡪 Overall Goodness of Information Provision (OGIP) | .50 [.37, .62] | <.001 | .39 |
| Organization-Wide Innovation Capability (IC OW) 🡪 Perceived HIT Workflow Support (PHITS) | .21 [.06, .36] | .01 | .06 |
| Organization-Wide Innovation Capability (IC OW) 🡪 Workflow Composite Score (WCS) | .03 [-.17, .22] | .78 | .01 |
| Clinical IT-Agents (CITA) 🡪 Workflow Composite Score (WCS) | .18 [.02, .33] | .02 | .06 |
| Moderating Effect (IC OW) 🡪 Overall Goodness of Information Provision (OGIP) | .21 [.08, .35] | .008 | .09 |
| Professionalism of Information Management (PIM) 🡪 Clinical IT-Agents (CITA) | .43 [.29, .57] | <.001 | .22 |
| Professionalism of Information Management (PIM) 🡪 Workflow Composite Score (WCS) | .48 [.31, .69] | <.001 | .28 |
| Structural Characteristics (SC) 🡪 Innovation Capability: Top Management Team Support (IC TMT) | .33 [.21, .43] | <.001 | .12 |
| Structural Characteristics (SC) 🡪 Professionalism of Information Management (PIM) | .35 [.24, .47] | <.001 | .39 |
| Structural Characteristics (SC) 🡪 Workflow Composite Score (WCS) | .13 [-.07, .31] | .19 | .02 |
| Workflow Composite Score (WCS) 🡪 Overall Goodness of Information Provision (OGIP) | -.06 [-.21, .07] | .33 | .01 |
| Workflow Composite Score (WCS) 🡪 Perceived HIT Workflow Support (PHITS) | .42 [.29, .54] | <.001 | .21 |
| Perceived HIT Workflow Support (PHITS) 🡪 Overall Goodness of Information Provision (OGIP) | .44 [.28, .58] | <.001 | .28 |
| Country (COU) 🡪 Innovation Capability of the IT Department (IC ITD) | .13 [.01, .25] | .03 | .03 |
| Country (COU) 🡪 Organization-Wide Innovation Capability (IC OW) | .22 [.10, .34] | <.001 | .08 |
| Country (COU) 🡪 Workflow Composite Score (WCS) | .14 [.01, .26] | .04 | .04 |
